# Physical Exercise, a Potential Non-Pharmacological Intervention for Attenuating Neuroinflammation and Cognitive Decline in Alzheimer’s Disease Patients

**DOI:** 10.3390/ijms23063245

**Published:** 2022-03-17

**Authors:** Samo Ribarič

**Affiliations:** Institute of Pathophysiology, Faculty of Medicine, University of Ljubljana, SI-1000 Ljubljana, Slovenia; samo.ribaric@mf.uni-lj.si

**Keywords:** Alzheimer’s disease, memory impairment, animal models, human studies, ageing, neuroinflammation, physical exercise

## Abstract

This narrative review summarises the evidence for considering physical exercise (PE) as a non-pharmacological intervention for delaying cognitive decline in patients with Alzheimer’s disease (AD) not only by improving cardiovascular fitness but also by attenuating neuroinflammation. Ageing is the most important risk factor for AD. A hallmark of the ageing process is a systemic low-grade chronic inflammation that also contributes to neuroinflammation. Neuroinflammation is associated with AD, Parkinson’s disease, late-onset epilepsy, amyotrophic lateral sclerosis and anxiety disorders. Pharmacological treatment of AD is currently limited to mitigating the symptoms and attenuating progression of the disease. AD animal model studies and human studies on patients with a clinical diagnosis of different stages of AD have concluded that PE attenuates cognitive decline not only by improving cardiovascular fitness but possibly also by attenuating neuroinflammation. Therefore, low-grade chronic inflammation and neuroinflammation should be considered potential modifiable risk factors for AD that can be attenuated by PE. This opens the possibility for personalised attenuation of neuroinflammation that could also have important health benefits for patients with other inflammation associated brain disorders (i.e., Parkinson’s disease, late-onset epilepsy, amyotrophic lateral sclerosis and anxiety disorders). In summary, life-long, regular, structured PE should be considered as a supplemental intervention for attenuating the progression of AD in human. Further studies in human are necessary to develop optimal, personalised protocols, adapted to the progression of AD and the individual’s mental and physical limitations, to take full advantage of the beneficial effects of PE that include improved cardiovascular fitness, attenuated systemic inflammation and neuroinflammation, stimulated brain Aβ peptides brain catabolism and brain clearance.

## 1. Introduction

The objective of this narrative review is to summarise the evidence for physical exercise (PE) to be considered as a non-pharmacological intervention for delaying cognitive decline in patients with Alzheimer’s disease (AD) not only by improving cardiovascular fitness but also by attenuating neuroinflammation.

First, the relevant background information is provided by introducing the subjects of PE, aetiology of AD, systemic low-grade chronic inflammation and ageing. Second, the interplay between the level of muscle work, AD-related changes that affect cognition, and inflammation is described at the level of molecular mechanisms. Third, recent relevant animal and human studies on the effect of muscle work on AD cognitive decline, indexed in PubMed between 2017–2022, are systematically presented. In addition to dominantly inherited familial or late onset animal models of AD, the animal model for cerebral amyloid pathology is also presented, since AD and cerebral amyloid pathologies coexist in some patients. The presented human studies published between 2017–2022, evaluating the effect of muscle work on attenuating cognitive decline, include randomised controlled trials, meta-analysis of randomised controlled trials, systematic reviews and meta-analysis of observational prospective studies and randomised controlled trials, and a prospective observational study. Only two randomised controlled trails, reporting the effect of muscle work on inflammation in patients with AD, were published between 2016–2022 and both are systematically presented. The review ends with an overall conclusion and suggestions for further work.

## 2. Physical Exercise

Muscle exercise is characterised either as physical activity (PA) or as PE. PA refers to any movement that is carried out by the muscles that requires energy. PE is a planned, structured, and repetitive PA with the objective to improve or maintenance of physical fitness. Physical fitness is a set of health or skill-related attributes that are evaluated by specific tests [1]. PE is preferred to PA for establishing the dose-related effects of muscle work on the human body.

The beneficial effects of regular, sustained PE (aerobic or resistance training) on the human body include:

(a) an increase in exercise tolerance (due to an increased cardiac and skeletal muscle strength, improved function and enhanced maximal oxygen consumption coupled with an increased capillary network);
(b) an increased insulin sensitivity in adipose tissue, skeletal muscle and endothelium leading to a reduced risk of systemic insulin resistance in persons type 2 diabetes;
(c) reductions in elevated body weight (due to an increased catabolism in muscles and adipose tissue) and blood pressure (due to an increased vascular density of arterioles and a reduction in systemic vascular resistance elicited by an increased release of vasodilatation promoting NO and prostacyclin from the vascular endothelium);
(d) an increase in HDL and LDL cholesterol particles size and a decrease in VLDL particles size and
(e) an improved response of the immune system with a delayed onset of immunesescence and reduced systemic inflammation (e.g., reduced numbers of exhausted/senescent T cells, an increased T-cell proliferative capacity, reduced blood levels of inflammatory cytokines, an increased neutrophil phagocytic activity, and an enhanced natural killer (NK) cell cytotoxic activity) [2,3,4].

The beneficial metabolic changes associated with regular PE are mediated by insulin-like growth factor 1 (IGF1) and insulin receptor signalling via the PI3K/AKT1/mTOR signalling that activate multiple transcriptional pathways [4].

The health effects of PE are enhanced when combined with optimal nutrition, secession of smoking and medication modification [2,3]. Sedentary persons have a considerably higher risk for cardiovascular disease than persons who engage in regular PE [4]. For example, a person who exercises about 5-times per week, has an approximately 50-times lower risk for cardiac related complications than a sedentary person [2]. Recommendations for a personalised, age-adjusted optimal amount of PE that sustains health benefits, have yet to be determined since high intensity PE can have detrimental health effects. For example, the relationship between PE workload and risk of upper respiratory tract infection follows a J-shaped curve. Compared to a sedentary life-style, a moderate PE workload reduces the risk of upper respiratory tract infection and a heavy PE workload is associated with an increased risk for upper respiratory tract infection [3].

Not all of the beneficial health effects of sustained PE (aerobic, resistance or concurrent exercise training) can be replicated in older adults. For example, a 12-week PE intervention improved cognitive function and physical fitness (evaluated by gait speed, upper and lower limb strength, aerobic fitness, hand-grip strength, timed up-and-go and sit-to-stand) in older adults (male and female, average age 69) compared to non-exercise control [5]. However, PE may not attenuate the decline in insulin sensitivity and muscle mass, or reduce blood pressure across all age groups. A recent study evaluated the effect of resistance PE on persons in young (age 18–28 years), middle-aged (age 45–55 years) and older (non-sarcopenic, age 65–75 years) cohorts after 20-weeks of supervised resistance PE. PE did not (a) attenuate baseline, age-related increased blood pressure values and increased adiposity in older individuals and (b) did not increase muscle mass in middle-aged and older individuals compared to their respective baseline values [6]. The effects of aerobic PE in older adults were reported to be sex dependent. Regular aerobic PE consistently improved the age attenuated endothelial function in men (by reducing oxidative stress and preserving NO bioavailability), but inconsistently in oestrogen-deficient postmenopausal women [7].

## 3. Alzheimer’s Disease

### 3.1. Aethiology, Risk Factors and Diagnosis

Alzheimer’s disease is the most common cause of cognitive impairment or dementia in individuals older than 65 years and rising global longevity is leading to a worldwide pandemic of mild cognitive impairment (MCI), AD, and AD-related dementia. There is no cure for AD; however non-pharmacological (e.g., exercise) and pharmacological (e.g., acetylcholinesterase inhibitors) interventions can mitigate the symptoms and attenuate the progression of the disease [8].

AD changes to conscious mental activity are not synonymous with old age changes. In AD multiple cognitive (e.g., attention, judgement, memory, intelligence, social cognition and executive function), functional and behavioural domains of conscious mental activity are impaired. In normal aging, there is a decline in fluid intelligence from early adulthood and a preservation and an increase in crystalized intelligence until late life. Also in normal aging, individuals retain at least some degree of their personality, interests, level of initiative, motivation, sociability, empathy and behaviour [9].

By aetiology, AD can be classified into three groups: dominantly inherited familial AD (FAD), early onset AD (EOAD) and late onset AD (LOAD). FAD (representing less than 1% of pathologically diagnosed AD cases, average age of onset 46 years) is caused by mutations in amyloid precursor protein (APP), presenilin-1 (PS1) or presenilin-2 (PS2) genes. EOAD (representing less than 5% of pathologically diagnosed AD cases) is present in patients with AD signs and symptoms before age 65. LOAD is the most common form of AD, where several genetic risk factors have been identified, including apolipoprotein (APO) ε gene, TREM2, ADAM10 and PLD3. Inheritance of APO ε4 also increases the risk for vascular dementia (VAD), Lewy body dementia (LBD), Down’s syndrome and traumatic brain injury. In summary, AD-risk attributable to genetic factors is estimated at 70%.

Ageing is the most important of all risks for development of AD [10]. For example, in the USA, in 2021, one in nine people aged 65 and older have AD dementia and almost two-thirds of Americans with AD are women. Brain blood flow is reduced with ageing, and the reduced blood flow and hippocampal volume are associated with a reduced cognition [11]. The documented changes of PE on the human brain are: improved cerebral brain blood flow, attenuated reduction in hippocampal volume and improved cognitive ability [12,13,14]. In addition to ageing, other nonmodifiable AD risk factors are cerebral amyloidosis, Down syndrome, gender (females have a greater AD risk), family history of AD and inheritance of APO ε4 allele [9].

Modifiable risk factors for AD can be stratified by age in three groups.: early life (ages below 45), midlife (ages 45–65) and later life (ages above 65). AD later life modifiable risk factors include diabetes, smoking tobacco and depression; midlife modifiable risk factors include hypertension, dyslipemia, metabolic syndrome and obesity. An example of an early life modifiable risk factor for AD is a low cognitive reserve [9].

An interesting risk factor for AD is late-onset epilepsy where perturbed inflammation is a shared pathogenesis factor by both brain disorders [15,16]. A recent study on 675 persons with epilepsy, and 2025 matched control subjects, reported that epileptic persons, age 50 years or above, have a greater risk of developing dementia than people without epilepsy [17]. Neuroinflammation is also a pathogenesis factor in other brain disorders, including Parkinson’s disease, amyotrophic lateral sclerosis and anxiety disorders [18,19].

In addition to AD, other common causes of dementia include VAD, LBD, Parkinson’s disease with dementia and frontotemporal lobar degeneration [9,20].

The key AD pathological features are the signs of a dual mixed proteinopathy, i.e., amyloid plaques and neurofibrillary tangles (NFT). Biochemical, neurophysiological, and neuroanatomical changes elicited by the AD dual mixed proteinopathy that can be measured decades before psychometrically and clinically noticeable deterioration in cognition, behaviour, and function. Therefore, AD dementia is a clinical diagnosis since, for example, 20–40% of individuals aged 70 or above do not have cognitive impairment in the presence of biomarkers for AD, or autopsy evidence of AD pathology. Also, AD-associated pathological brain changes often coexist with other pathologies, for example with VAD, LBD or cerebral amyloid angiopathy and these comorbidities, in conjunction with the presence of modifiable risk factors, contribute to the variety of AD clinical signs and symptoms that increase the complexity for development of effective diagnostic tools and treatment interventions [8,9,20].

The preclinical stage of AD (with normal cognition), i.e., the time before development of AD-related clinical signs and symptoms, can last for decades. At present, the preclinical stage of AD can be detected with a combination of complimentary biomarkers in cerebrospinal fluid (Ab42, tau, phosphor-tau), non-invasive neuroimaging, and genetic evidence of AD. Future, AD biomarkers are being developed on the basis of functional MRI, diffusion tensor imaging MRI, arterial spin labelling MRI, and advanced PET [21,22,23].

Therefore, due to the long duration of the preclinical stage of AD, only interventions that are effective, sustainable, with a high patient compliance, and with a few side effects for decades can contribute to a measurable delay in the development of AD-related signs and symptoms [24]. PE can ameliorate the negative effects of physical inactivity that is a very common, and also a preventable risk factor for AD [10]. Fewer side effects and a better patient compliance are the key advantages of PA over medications [25]. Therefore, PA could be useful for AD prevention and for ameliorating the early stages of AD [26].

### 3.2. AD Antimicrobial Aβ Peptides and Tau Protection Hypothesis of AD

The hallmarks of AD are (a) the increased brain expressions of per se normal Aβ and tau, peptides with known physiological brain functions [27,28], and (b) formation of early-stage toxic monomers, oligomers, late-stage fibrils and β-amyloid from Aβ peptides, and NFT from phosphorylated tau. These perturbations of tau and Aβ proteostasis are associated with chronic neuroinflammation (where the release of pro-inflammatory molecules is favoured over the release of anti-inflammatory molecules), progressive reduction of brain synapses, reduction of neurons and progressive cognitive decline. The relevance of perturbed Aβ and tau proteostasis has been substantially documented in early-onset AD patients [29] and in animal models where Aβ and tau peptide were overexpressed [30]. About 95% of the patients with AD have a late-onset form without an identified mutation in Aβ nor in tau proteostasis [31,32]; also, numerous studies have identified non-genetic risk factors for AD [33].

An essential function of Aβ42, relevant to the pathogenesis of AD in human, is its role in the innate immune response to bacterial, fungal or viral infections, studied in human and animal models [34,35,36]. The propensity of Aβ42 for oligomerisation and β-amyloid formation is assumed to be a cross-species conserved ability for pathogen entrapment and containment of brain infection [37,38,39]. Agglutination and entrapment activities, preventing the binding of pathogens to host cells, were attributed to Aβ oligomers, protofibrils and fibrils that bind pathogens within a protease-resistant amyloid matrix [40,41]. Discoveries related to the role of Aβ peptides in innate immunity were integrated into the “Antimicrobial Protection Hypothesis” of AD (APH-AD). The APH-AD attributes the development of late-onset, human AD to the perturbed response of the innate immunity where the normal, short term-acute immune response to brain pathogens is sustained in the long term even in the absence of infection, especially in the presence of some nongenetic risk factors that also promote/sustain the inherent Aβ peptide self-aggregation (e.g., long-term systemic inflammation associated with diabetes type 2) [33]. In physiological conditions, the APH-AD model predicts a two-step innate immune response to pathogen infection: entrapment of the pathogen by Aβ peptide, associated by a transient, short-term, activation of pro-neuroinflammatory pathways in the brain, followed by a shift in the balance of neuroinflammatory pathways that favours anti-inflammatory pathways and clearance of Aβ peptide self-aggregation products (monomers, oligomers, protofibrils, fibrils and amyloid) from the brain. Some Aβ products (oligomers, protofibrils and fibrils) act as nucleation sites, therefore their timely removal is vital to prevent amplification of their production and sustained neuroinflammation that potentially could persist in the absence of brain pathogens [42,43].

In AD, there is a sustained activation of Aβ peptide production and self-aggregation, associated with brain neurodegeneration, where neuroinflammation drives the positive feedback-loop of Aβ products and neurodegeneration. Also, β-amyloid overexpression promotes formation of NFT from endogenous tau [34,44] further supporting brain neuroinflammation and neurodegeneration. The APH-AD is supported by experimental evidence, summarized below:

(a) some elderly persons with widespread Aβ products deposition and NFT, and with no signs of dementia at the time of death, do not have brain gliosis and neuroinflammation [45];
(b) attenuation of pro-inflammatory immune pathways reduces Aβ products deposition [46];
(c) in human, pharmacologically reduced Aβ peptide is associated with an increased risk of infection [39,47];
(d) anti-viral drug treatment of infected patients reduced the risk for development of dementia compared to untreated, infected patients [48,49];
(e) APO ε gene expression promotes a pro-inflammatory innate immune response, with APO ε4 allele having the strongest [50,51];
(f) the majority of soluble amyloid β oligomers (AβO) have a broad-spectrum of antimicrobial activity associated with a variety of oligomer post-translational modifications, essential for oligomerisation and antimicrobial properties, and with no neurotoxic effects [52,53];
(g) Aβ is an anionic antimicrobial peptide (A-AMP); Aβ42 and Aβ42 products (e.g., AβO) have neurotoxic and antimicrobial effects that elicit disruption of cell and mitochondrial (MITO) membranes; in contrast to cationic AMP, A-AMPs are more likely to bind to eukaryotic, bran cell membranes, however, they are less susceptible to proteases secreted by bacteria when entrapped with AMPs and thus more effective against microbes then cationic AMP [54,55,56,57];
(h) Aβ42 and their AβOs simultaneously bind Zn^2+^ or Cu^2+^ ions and these bindings enhance their specificity and affinity for microbial cell membranes; presumably, some subtypes of the post-translationally modified Aβ42 products have the lowest affinity for Zn^2+^ or Cu^2+^ ions and thus the highest affinity for brain cell membranes, are the most neurotoxic [52];
(i) brains of patients with AD, have a higher level of brain microbial/vial pathogens burden (e.g., in the hippocampus), compared to normal control brain tissue, and inheritance of the APO ε4 allele increases the risk for both late-onset AD and central nervous system (CNS) infection [58];
(j) overexpression of neuroinflammation associated microglial genes is a risk factor for late onset AD [59,60] and impaired microglial chemotactic and phagocytic functions promote brain Aβ peptide deposition [61];
(k) increased levels of interferon-induced transmembrane protein proteins, in human with late-onset Alzheimer’s disease or in animal models of AD, due to an increased release of pro-inflammatory cytokines from neurons and astrocytes in response to viral infections and/or ageing, have a dual effect: they attenuate viral cell entry by reducing cell membrane fluidity of viral fusion sites, thus preventing viral fusion pore formation, however, they also enhance production of Aβ40, Aβ42 and β-amyloid by binding and increasing the activity of γ-secretase [34,62];
(l) infection of primary adult rodent hippocampal neuronal cultures with Herpes simplex virus 1 elicited a dual response: a transient increase in tau protein content and a long term, persistent increase in Aβ42 products deposition [63].

Tau contributes to normal cytoskeletal stability, axonal development, intracellular trafficking and synaptic plasticity. However, phosphorylation of tau by Fyn enables localisation of Fyn tyrosine kinase to dendritic spines, where the Tau/Fyn complex associates with the postsynaptic density-95 scaffolding protein to enable phosphorylation of the N-methyl-D-aspartate receptor and ion channel (NMDAR) subunit NR2B with Fyn. The successful formation of the NR2B/PSD-95/tau/Fyn complex opens the NMDAR, promoting Ca^2+^ influx, intracellular Ca^2+^ overload and neurotoxicity. The hallmark of AD is amyloid-β deposition in the brain. AβO bind to the NR2A subunit and activate the NMDARs promoting intracellular Ca^2+^ influx, followed by either intracellular Ca^2+^ overload and neuronal death in the extreme, or synaptic NMDAR desensitization, NMDAR internalization and long term depression of synaptic transmission [64]. This AβO toxicity is tau dependent and the contribution of tau phosphorylation to the development of Aβ toxicity is site specific. T205 phosphorylation, activated by p38 mitogen-activated protein kinase p38g, inhibits the formation of postsynaptic excitotoxic signalling complexes NR/PSD- 95/tau/Fyn and Aβ toxicity [65].

### 3.3. Brain Aβ Peptide Clearance Is Dependent on Aβ Peptide Catabolism in Peripheral Organs

Aβ peptide metabolism is present not only in the brain cells, but also in the peripheral organs and tissues. For example, AβPP is expressed in liver, kidney, heart, pancreas, spleen, skeletal muscle and blood cells (e.g., platelets). However, in AD, Aβ peptides are only deposited in the brain and not in peripheral organs of tissues. The dominant Aβ peptide in the plasma is Aβ40, and Aβ42 in the brain. The concentrations of AβPP and Aβ peptides are 10-times lower in the plasma than in the CSL due to a shorter half-life of AβPP and Aβ peptides in the plasma (promoted by binding to lipoproteins and albumin). The catabolism of Aβ peptides in the brain is accomplished by enzymatic intracellular (IDE, ubiquitin-proteasome pathway and lysosomal cathepsin enzymes in neurons) and extracellular pathways (e.g., NEP degrades Aβ peptides and, IDE degrades Aβ peptides), or non-enzymatic pathways (receptor or non-receptor mediated uptake by neurons, microglia, astrocytes, perivascular macrophages, and oligodendroglia). The brain neurons are the predominant cells for Aβ peptide synthesis and degradation. Clearance of Aβ peptides from the brain to the periphery, over the blood-brain barrier (BBB), is by the low-density lipoprotein receptor-related protein, receptor for advanced glycation products and ABC transporter-mediated clearance. Also, the BBB uptakes and catabolises brain and periphery derived Aβ peptides. About 60% of brain derived Aβ peptides cross the BBB and are catabolised in peripheral organs with the bulk of catabolism in the liver and kidney [66].

Chronic diseases alter Aβ peptide metabolism. For example, (a) the IDE mediated catabolism of brain Aβ peptides is attenuated by diabetes mellitus (DM) hyperinsulinemia and hyperglycaemia; (b) the high cell membrane cholesterol content stimulates β- and γ-secretase activity; (c) chronic heart failure attenuates clearance of Aβ peptides, over the BBB, from the brain to the blood and catabolism in the peripheral organs and tissues and (d) liver failure significantly attenuates Aβ peptides clearance from the blood (90% of circulating Aβ peptides are removed from the blood by hepatocytes) [66].

## 4. Ageing

### 4.1. Ageing Is Associated with Systemic Low-Grade Chronic Inflammation

Human ageing, the most important of all risks for development of AD, is associated with a gradual transition of an acute inflammatory response to a systemic low-grade chronic inflammation (SCI). SCI is triggered by damage associated molecular patterns (DAMPS, e.g., non-encapsulated MITO DNA, dysfunctional organelles, misfolded and/or oxidized endogenous proteins, mRNA from necrotic cells, heat shock proteins, high mobility group box 1, Aβ peptides and oligomers) in the absence of an interaction between pattern recognition receptors (PRRS) on innate immune cells and evolutionarily conserved pathogen-associated molecular patterns (PAMPS). The presence of SCI in older individuals is evidenced by increased blood levels of pro-inflammatory cytokines, chemokines and acute phase proteins and an increased expression of pro-inflammatory genes that ultimately accelerate ageing by stimulating the development of chronic disorders, reduced general health and lifespan. For example, the pro-inflammatory cytokine tumour necrosis factor α (TNFα) promotes insulin resistance in adipocytes which leads to development of diabetes type 2 and an increased risk for diabetes type 2 promotes cardiovascular neurodegenerative, cancer and autoimmune diseases [67,68,69,70].

When the effects of SCI spread to numerous organs (e.g., to the brain, gut, liver, kidney, adipose tissue and muscle) this condition is called inflammaging. The inflammaging process, the long-term result of the chronic stimulation of the innate immune system in old age, modulates multiple interconnected biochemical molecular pathways including adaptation to stress, epigenetics, inflammation, macromolecular damage, metabolism, proteostasis, stem cell and tissue regeneration [71].

Inflammaging is triggered by a combination of an inappropriate initiation of inflammation and an inadequate resolution of inflammation (possibly due to an attenuated macrophage phagocytosis of apoptotic cells when there is a lack of pro-resolving mediators (e.g., resolvins, protectins or maresins) to polarise these cells to a pro-resolution phenotype). This ageing associated inflammation process is sustained by persistent tissue damage which results in further inflammation and additional tissue damage [72]. The risk for SCI and inflammaging is increased by maternal inflammation during pregnancy and childhood obesity [67]. Persons with SCI or inflammaging have an increased susceptibility to viral infections and a weakened response to vaccines due to a diminished immune response to acute challenges elicited by a reduced phosphorylation of signalling proteins in the cytokine-activated Janus kinase (JAK)—signal transducers and activators of transcription (STAT) signalling pathway [67].

The long-term effect of SCI is the development of inflammation-related diseases (e.g., DM and neurodegenerative disorders) with a reduced quality of life and an increased risk of mortality [67]. Chronic inflammatory diseases are the most significant cause of death worldwide [73].

The association between SCI and increased risk of inflammation-related diseases is supported by randomised controlled trials that evaluated the effect of anti-inflammatory interventions on inflammation-related diseases [67]. For example, in patients with rheumatoid arthritis and diabetes, anti-TNFα inhibitor therapy attenuated the patients’ insulin resistance and improved insulin sensitivity [67], also the risk for developing Alzheimer’s disease was reduced [67]. However, biomarkers to detect and evaluate the effect of SCI on inflammation-related diseases have yet to be developed [67].

The transition of an acute inflammatory response to a SCI is facilitated by social, psychological, environmental and biological factors that prolong the normal, temporally restricted inflammation and change the expression pattern of pro-inflammatory molecules (e.g., IL1β, IL6, TNFα, monocyte chemoattractant protein-1) and participating immune cells [67]. The development of SCI is not fully understood. Senescence of immune cells, i.e., the development of a senescence-associated secretory phenotype (SASP), is assumed to be an important contributing factor, evidenced by an increased secretion of pro-inflammatory molecules that promote inflammation-related diseases [67].

Biochemical molecular pathways associated with DNA damage, dysfunctional telomeres, epigenomic disruption and oxidative stress in combination with factors (chronic infections, lifestyle-induced obesity, microbiome dysbiosis, diet, social and cultural changes and environmental and industrial toxicants) seem to contribute to the development of SASP in senescent cells. The expression of SASP depends on inter-individual differences in exposure, their genetic predisposition and general health.

Environmental factors were identified as key drivers of SCI [67]. Examples of factors promoting SCI are:

(a) chronic infection with human immunodeficiency virus with accumulation of senescent CD8+ T cells responsible for increased levels of pro-inflammatory molecules [67];
(b) a low PA, associated with a reduced release of cytokines and myokines from skeletal muscle cells during contraction, reduces the positive effect of these muscle molecules on attenuating systemic inflammation [67] and promotes the development of type 2 diabetes, sarcopenia, depression, and different types of dementia including Alzheimer’s disease [67];
(c) an excessive increase in visceral adipose tissue (VAT) mass (due to adipocyte hypertrophia and/or hyperplasia), associated with a low PA and an inappropriate diet, promotes local hypoxia and increased activation of hypoxia-inducible factor1α, production of reactive oxygen species, and release of DAMPS followed by an increased secretion of pro-inflammatory molecules and chemokines by VAT adipocytes, endothelial cells and resident macrophages [74] which elicits an infiltration of VAT with additional immune cells, (i.e., monocytes, neutrophils, dendritic cells, B cells, T cells and NK lymphocytes, and a concomitant reduction in T regulatory cells); the overall effect is an enhanced VAT initiated inflammation and transition of this local inflammation to a SCI [67];
(d) microbiome dysbiosis (e.g., changes in gut microbiota composition and gene pool, increased intestinal paracellular permeability and endotoxemia), is associated with multiple causative factors including overuse of drugs, lack of microbial exposure during or after birth, diabetes type 2, and obesity may lead to or sustain SCI [67];
(e) a diet rich with processed food (with a high fat, sugar, salt and additives content and low on fresh fruits, vegetables, fibber content, micronutrients and long chain omega 3 fatty acids) is associated with SCI and microbiome dysbiosis [67]; examples are high-glycaemic-load foods common in processed food that promote activation of oxidative stress pathways that activate pro-inflammatory genes [67] and deficient intake of long chain omega 3 fatty acids, due to a diet of mainly processed foods, that reduces the human body’s ability to form inflammation attenuating molecules (e.g., resolvins, maresins and protectins) [67];
(f) industrial toxicants (e.g., phthalates, bisphenols, polycyclic aromatic hydrocarbons and flame retardants1) promote inflammation, for example via oxidative stress, and increase the risk for neurodegenerative diseases, type 2 diabetes and metabolic syndrome among others [67].

### 4.2. Molecular Mechanisms of Inflammaging

Molecular mechanisms that initiate and sustain inflammageing include: a decreased protein kinase B (AKT) activation and an increased glycogen synthase kinase 3β (GSK3β) activity [75]; an increased reactive oxidative species (ROS) production [76]; chronic elevated glucocorticoid (GCs) levels [75]; a chronic activation of pro-inflammatory transcription factor NFκB [75]; a defective mitophagy and mitochondria biogenesis, an insulin resistance [76]; an increased catabolism in muscle (development of sarcopenia) and nerve cells (attenuated neurogenesis and plasticity) [76]; a reduced IGF1 synthesis and activity [76]; and an increased rate of inflammasome activation [77].

Pro-inflammatory cytokines IL1α, IL6, and TNFα reduce IGF1 synthesis and activity [78], this growth factor is essential for the maintenance of muscle strength and regeneration, thus increasing the risk for sarcopenia, accelerated ageing and reduced life span. TNFα also activates NFkB that promotes gene expression of pro-inflammatory and proliferative proteins and also inhibits, at the post-transcriptional level, skeletal muscle differentiation [79]. Inflammation also reduces the muscle’s ability to adjust the rate of perfusion to the rate of muscle work, thus favouring muscle protein catabolism over anabolism [76].

The production of pro-inflammatory cytokines and enhanced protein catabolism are sustained by defective mitophagy that prolongs the production and release of ROS from dysfunctional mitochondria into the intracellular and extracellular space [76]. Insulin resistance, elicited by the binding of agonists to tumour necrosis factor receptor superfamily member 1A (TNFR1) and toll-like receptor 4 (TLR4), activate the JAK signalling pathway that phosphorylates serine at insulin receptor substrates 1 and 2 (IRS1, 2) [76]. TNFα, IL1β, and IL6 disrupt MITO biogenesis as evidenced by a reduced adenosine triphosphate (ATP) synthesis, reduced nicotinamide adenine dinucleotide (NAD^+^):NADH ratio, and reduced mRNA levels of *PPARGC1A* encoding peroxisome proliferator-activated receptor-γ coactivator (PGC1α) [80]. Ageing increases the probability of inflammasome activation, thus increasing the rate and duration of pro-inflammatory responses, and development of inflammaging. Inflammasomes activate the innate immune system inflammatory response. Increased inflammasome activation is attributed to aging microglia (i.e., to aging macrophages resident in the CNS with altered cytokine production due to an increased state of oxidative stress) that, with ageing, are more likely to be primed for pro-inflammatory responses then for inflammation resolution responses. Presumably the local microenvironment changes in the brain (e.g., an increased TNFα activity) or systemic changes in the body (e.g., systemic infections) are responsible for initiating and/or sustaining metabolic changes in the aging microglia [77].

### 4.3. Inflammaging and Neuroinflammation

Brain neural activity is supported by glial cells: microglia—resident macrophages (responsible for pruning synapses, regulating neural death and waste elimination), astrocytes and oligodendrocytes. Astrocytes support normal neuronal activity by maintenance of the BBB, by providing neurons with energy and metabolic substances and also modulate neuronal excitability, synaptic development and action potential transmission. Microglia and macrophages (from blood, that cross the BBB) clear cellular debris in the diseased CNS. Oligodendrocytes provide support and insulation to axons by creating myelin sheaths; a single oligodendrocyte provides myelin sheaths to axons of several neurons. In physiological conditions the crosstalk among astrocytes and microglia on the one hand and oligodendrocytes on the other supports the development of oligodendrocyte progenitor cells into mature myelinating oligodendrocytes and provides for myelin maintenance on neurons. [68].

Microglia and astrocytes contribute to the innate immune response in the CNS which is triggered by contact of PRRS (TRL2 and 4) on these cells with PAMPS (in CNS infections) or DAMPS (in CNS neurodegenerative diseases). The innate immune response includes proliferation and migration of astrocytes, release of inflammatory molecules from astrocytes and microglial cells, clearance of extracellular aggregates via phagocytosis and endocytosis, CNS infiltration of leukocytes across the BBB due to a vascular endothelial growth factor (VEGF) increased permeability and astrocytes chemokine triggered recruitment. Contact between PRRS and PAMPS or DAMPS transforms astrocytes into A1 reactive astrocytes. During neuroinflammation the proportion of pro-inflammatory A1 reactive astrocytes (capable of phagocytosis and secretion of IL1, IL6, TNFα, CC motif or CXC motif, ROS, glutamate and VEGF) increases compared to the A2 reactive astrocytes fraction (secreting anti-inflammatory molecules). The proportion of A1 astrocytes is significantly increased in normal ageing and in neurodegenerative diseases (e.g., AD) [81,82,83].

Inflammaging increases the risk for neurodegenerative diseases, where ageing of both innate and adaptive immune responses has an important role. Macrophages and microglia display signs of impaired and prolonged activation to PAMPS or DAMPS, reduced motility and impaired phagocytosis [84]. Excessive release of glutamate and ROS contribute to neural and oligodendrocyte death [68] and a persistent accumulation of misfolded/modified Aβ peptides and Aβ oligomers degrades astrocyte function and precipitates cell death [85,86]. Increased release of TNFα from A1 reactive astrocytes and activated microglia promotes amyloid plaque formation by increasing PS1 and β-secretase synthesis. TNFα genetic deletion in 5XFAD mice attenuates amyloid plaque formation by lowering Aβ content via reduced PS1 and β-secretase production [87]. Astrocyte cell death and/or chronic release of inflammatory cytokines from microglia and astrocytes contributes to demyelination or attenuated remyelination of neurons and consequent functional changes in brain neuronal networks [68,84]. Systemic infections could contribute to the development and progression of neurodegenerative diseases by priming the CNS’s innate immune response. For example, patients with AD have an increased proportion of pro-inflammatory gut bacteria compared to control [88]. Inflammaging also attenuates brain neurogenesis, and reduced neurogenesis contributes to development of neurodegenerative diseases [89].

### 4.4. Ageing Reduces the Efficienty of Innate and Adaptive Immunity Responses

Ageing-related changes affect the innate and adaptive immunity responses. Changes to the innate immunity are:

(a) an increased lifespan of macrophages due to sustained stimulation with PAMPS that bind to TLR (e.g., LPS), attenuated chemotaxis, superoxide production and expression of TLR in macrophages;
(b) increased numbers of NK and NKT cells with reduced per-cell cytotoxicity and cytokine production;
(c) increased levels of pro-inflammatory cytokines IL1, IL6 and TNFα in the extracellular space; and
(d) reduced numbers, distribution, migration and MHC expression and signalling in dendritic cells. Immunosenescence-associated changes to the adaptive immunity were observed in B cells (reduced number and capacity for antibody production to new antigens) and T cells (increased number of memory cells, regulatory T cells, CD28 cells, release of Th1 cytokines; and reduced numbers of naive T cells, reduced CD4:CD8 ratio, reduced proliferation, release of Th1 cytokines, cytotoxicity, and T cell receptor variety) [90,91].

Immunosenescence is associated with (a) an impaired proteasome degradation linked with impaired antigen presentation; (b) an increased intracellular accumulation of misfolded protein aggregates (e.g., AGE, heat shock proteins)—due to ageing combined with chronic inflammatory conditions (e.g., diabetes mellitus)—that promote cell death; (c) an increased release of pro-inflammatory cytokines to the plasma from immune cells (macrophages, B and T lymphocytes and mast cells) and non-immune cells (e.g., endothelial cells, fibroblasts); and (d) increased levels of DAMPS (released by cell death, and/or an increased release of gut microbial products into the blood, due to an increased gut permeability) that elicit a sustained response of innate immunity by activation of the nuclear factor kappa-light-chain-enhancer of activated B cells (NFκB) pathway and NLR family pyrin domain containing 3 protein (NLRP3) inflammasome, both contributing to a low-grade, sterile inflammation [90,91,92,93].

Key drivers, that modify the immune system during ageing, are sustained and increased ROS production with release into the extracellular space and long-term increased levels of post-translationally modified proteins (e.g., oxidised LDL, AGE and DAMPS). For example: (a) oxidised LDL activate TLR8 and TLR2 signalling pathways; (b) AGE and DAMPS bind to receptor for advanced glycation end products (RAGE) promoting long-term activation of the NFκB signalling pathway; and (c) increased levels of ROS, released by immune cells, promote NLRP3 inflammasome formation and increased levels of pro-inflammatory cytokines [91].

### 4.5. Ageing and Obesity Modulate the Innate and Adaptive Immune Responses

Obesity attenuates antibody responses in individuals of all age groups; obese persons have a ratio of anti-inflammatory B lymphocytes (subset of transitional cells) to pro-inflammatory B lymphocytes (late/exhausted memory cells subset) that favours the latter. Ageing increases total human body adiposity and also changes the ratio of VAT to subcutaneous adipose tissue (SAT) in favour of VAT that secretes more pro-inflammatory mediators (e.g., adipose tissue derived leptin) compared to SAT. High levels of leptin are associated with ageing and obesity (an increased body VAT). Leptin stimulates: (a) secretion of pro-inflammatory cytokines from macrophages; (b) production and secretion of pro-inflammatory cytokines (e.g., TNFα) in B lymphocytes, and life-span extension by inhibiting apoptosis of B cells; (c) inhibition of regulatory T cells; and (d) activation of T helper type 1 (Th1) CD41 T cells that infiltrate VAT, promote secretion of pro-inflammatory cytokines from M1 macrophages thus contributing to local and systemic inflammation. Also, adipocytes secrete chemokines that promote migration of B lymphocytes to VAT and pro-inflammatory cytokines that contribute to systemic chronic inflammation [94].

## 5. Chronic Neuroinflammation Has a Significant Impact on the Initiation, Sustainability and Progression of AD

### 5.1. Overview

SCI risk factors, promoting systemic inflammation, also contribute to neuroinflamation, since pro-inflammatory cytokines (e.g., TNFα, IL1) cross the BBB. Neuroinflammation induces, sustaines and accelerates neurodegeneration in Parkinson’s disease, Alzheimer’s disease and multiple sclerosis [95,96].

Chronic, low grade neuroinflammation, as part of the bodies systemic inflamaging, has a significant impact on the initiation, sustainability and progression of AD. Ageing accelerates diverse intracellular changes, including attenuated DNA damage repair with MITO dysfunction (especially in postmitotic tissues—e.g., the brain—with a high level of oxidative stress and a reduced nuclear and MITO DNA repair capacity), telomere shortening, loss of proteostasis, altered intercellular communication with deregulated cellular metabolism (e.g., due to lack of intracellular NAD^+^) and stem cell exhaustion. These intracellular changes are reflected in senescent neurons, astrocytes, microglia and oligodendrocytes, associated with chronic, low grade neuroinflammation [77,97]. Senescent brain cells propagate the oxidative and inflammatory stress to neighbouring, normal cells and induce in them a senescent phenotype [98,99].

Aggregating Aβ peptides stimulate oxidative and inflammatory stress in a cell culture model [100]. Brain tissues of patients with AD have an increased expression of the senescence- associated β- galactosidase activity [101]. In addition to the TRL pathway, the inflammatory response in brain cells can be triggered via the Notch receptors or inflammasomes. The pro-inflammatory phenotype of senescent astrocytes, i.e., increased production of inducible nitric oxide synthase (iNOS), IL1b, IL6, and TNFα, can be activated by the Notch—phosphatidylinositol 3-kinase—AKT signalling pathway, an alternative pathway to TRL, for activating an inflammation response in astrocytes [102]. Increased NLRP3 gene expression promoted inflammasome activation in microglia cells, with increased production of pro-inflammatory cytokines IL1β and IL18 [77]. Transplantation of gut microbiome from a patient with AD into (a) wild type mice and (b) AD model mice increased NLRP3 expression in the intestinal tract of the recipient animals in both groups, with concomitantly increased peripheral blood levels of pro-inflammatory cytokines. The recipient wild type mice had an increased expression of pro-inflammatory cytokines in microglia of hippocampi, without signs of a significant cognitive impairment; the recipient AD model mice had a more severe cognitive impairment compared to control, transplant-free AD model mice [103]. During ageing, pro-inflammatory cytokines IL1β and TNFα, released during chronic low-level inflammation, cross the BBB and prime microglia and astrocytes to favour proliferation, phagocytosis and the release of pro-inflammatory cytokines over the release of anti-inflammatory cytokines when responding to a direct, secondary pro-inflammatory stimulus (e.g., misfolded α-synuclein and fibrillar Aβ). This priming process of microglia and astrocytes is amplified in AD and other neurodegenerative disorders [104,105,106]. TNFα or Aβ oligomers/fibrils stimulate NLRP3 gene expression and inflammasome activation in microglia cells that promotes further formation of tau and Aβ toxic aggregates [107,108,109,110,111].

### 5.2. Increased GSK Activity Promotes Chronic Neuroinflammation and AD Etiology

Brain GSK3β is involved in many, key nerve cell signalling pathways including MITO energy metabolism, neurogenesis, neuronal migration, neuronal polarization, and axon growth and guidance among others. Therefore, the activity of this enzymes is highly controlled by inhibitory phosphorylation by protein kinase A, protein kinase B and protein kinase C [112,113]. Ageing is associated with an increased activity of GSK3β, a sign of declining GSK3β homeostasis [75,114].

GSK3β dysregulation, an increased and sustained activation of this signalling pathway, initiates and promotes chronic neuroinflammation and contributes to development of AD brain pathology (excessive tau phosphorylation and formation of NFT, excessive amyloid-β production and production of toxic monomers and oligomers) and accelerated cognitive decline by inhibiting neurogenesis, synaptic function and memory formation [115].

GSK3β substrates in the amyloidogenic pathway are β-secretase (BACE1) and PS1, (a catalytic component of γ-secretase complex), thus shifting the balance of APP cleavage from the non-amyloidogenic pathway, where APP is sequentially cleaved by α- and γ-secretase complexes to rapidly degraded peptides, towards the amyloidogenic, Aβ (40–42) producing pathway, prone to toxic peptide oligomerisation and fibril formation [116,117,118,119].

Excessive GSK3β activation attenuates the activity of the PS1/N-cadherin/beta-catenin complex, thus reducing synaptic function and promoting nerve death [119,120]. GSK3β modulates the balance between long term potentiation (LTP) and long-term depression (LTD). GSK3β overactivation promotes NMDA receptor-dependent LTD over the GSK3β inhibited AMPA receptor-dependent LTP, leading to attenuated memory formation and learning [121,122]. In addition, GSK3β overactivation promotes β-catenin phosphorylation and proteasome degradation; elimination of β-catenin from the pre- and post-synaptic membranes reduces the number of synaptic connections [123,124,125].

BACE-1 overactivation with GSK3β is mediated by NFκB signalling, thus establishing a link between pro-inflammatory signalling pathways and GSK3β overactivation. Both GSK3β and NFκB are overexpressed in patients with AD [126,127]. Inhibition of GSK3β, in a cell model of AD, attenuated BACE1 cleavage of APP and production of Aβ (40–42) peptides [128,129].

GSK3β activity regulates the balance between pro-inflammatory and anti-inflammatory mediators in immune cells of the human body. Increased brain GSK3β activity (a) up-regulates the release of pro-inflammatory molecules; (b) down-regulates the release of anti-inflammatory molecules (interleukin (IL) -1Ra, -4, -10, and transforming growth factor β); (c) inhibits phagocytosis of Aβ; and (d) promotes transformation of naive CD4+ T cells to Th2 cells that contribute to chronic inflammation [112]. Since the Aβ phagocytosis capacity of microglia is limited, GSK3β overactivation further reduces the microglial ability to prevent accumulation of Aβ toxic products [112]. Therefore, GSK3β overactivation in the brain is associated with an increased release of pro-inflammatory cytokines (IL -1, -6, -8 and TNFα) from astrocytes and microglia, promoting a neurotoxic environment and nerve cell death [115]. GSK3β overactivation promotes an increased release of pro-inflammatory cytokines by enhancing c-Jun N-terminal kinase (JNK), STAT3/5 and NFκB signalling and also promotes microglia migration [112,130,131]. The products of GSK3β overactivity, Aβ toxic monomers, oligomers and fibrils, also stimulate the release of pro-inflammatory cytokines and ROS/reactive nitrogen species from microglia thus establishing a self-propagating cycle of chronic neuroinflammation [112]. AβO also bind to nerve cell α2A adrenergic receptors that amplifies, by two orders of magnitude, GSK3β activation and tau hyperphosphorylation in response to very low, nanomolar accumulations of extracellular Aβ peptides. The activity of α2A adrenergic receptors was reported to be elevated in patients with AD and AD mouse models [132].

Increased GSK3β brain activity, observed in animal models of diabetic mice, was associated with a reduced IDE activity and increased Aβ peptide levels in the brain [116,133,134,135,136]. Increased levels of Aβ peptides (a) directly activate GSK3β activity and consequent GSK3β mediated tau phosphorylation with accelerated toxic, tau self-aggregation and (b) attenuate the Wnt pathway inhibition of GSK3β activity, thus further accelerating the development of Aβ and tau pathology [116,137,138,139].

Dysregulation of GSK3β activity is associated with the loss of hippocampal and basal forebrain cholinergic neurons. Overactivation of GSK3β is assumed to decrease acetylcholine brain levels due to altered intracellular distribution of choline acetyltransferase activity in selected cholinergic neurons. Stimulation of cortical, striatal and hippocampal cholinergic pathways reduced GSK3β activity [116].

MITO energy metabolism is regulated by GSK3β activity. Aging is associated with an increased GSK3β activity and decreased hippocampal PGC-1α protein levels. GSK3β inhibition stimulated MITO energy metabolism, as evidenced by increased MITO proton motive force and concomitant increased MITO respiration. This improved MITO respiration was associated with an increased PGC-1α activity (a measure of increased MITO biogenesis) and improved nuclear localisation. Therefore, the aging related increase of GSK3β activity could contribute to cognitive impairment, elicited by MITO dysfunction, in the early stages, pre-clinical stages of AD [113].

### 5.3. Does Excessive Endoplasmic Reticulum Stress Promote Chronic Neuroinflammation and AD?

ER responds to (a) increased ROS, protein production/availability (e.g., due to tau hyperphosphorylation), or to (b) mutant (mutant PS2 or tau overexpression), unfolded or misfolded proteins (e.g., oxidised Aβ, AβO and S-nitrosylation of molecular chaperons, MITO proteins, and synapse proteins in AD) with activation of the unfolded protein response (UPR). The UPR facilitates ER-associated protein degradation (ERAD) in a high Ca^2+^ and oxidising environment. The UPR’s response depends on the magnitude and duration of ER’s stress. In the short term and in the presence of a moderate ER stress, UPR triggers molecular pathways that stop protein translation, promote degradation of misfolded proteins and stimulate synthesis of protein folding molecular chaperons. In the presence of severe ER stress (increased cytosolic ROS and Ca^2+^, reduced ATP production in MITO) or moderate intensity, long-term stress that leads to further dysregulation of ER folding, maturation, trafficking and quality control of synthesized proteins, UPR triggers autophagy or apoptosis [105,140,141].

Three activated ER’s transmembrane proteins, IRE-1 (inositol requiring protein 1), protein kinase R-like endoplasmic reticulum kinase (PERK) and ATF6 (activating transcription factor 6), mediate the UPR. Phosphorylated IRE-1 upregulates the expression of ERAD associated enzymes and ER chaperones, to restore normal ER proteostasis. PERK inhibits overall protein synthesis by attenuating ribosome assembly and lowers the ER protein burden. ATF6 promotes the expression of ERAD associated chaperones and protein folding enzymes [105]. In mammals, there is a positive signalling loop between ER stress response and inflammation. In ageing, as well as in AD, diabetes and obesity, all three UPR signalling pathways trigger and sustain the production of microglial and astrocytes pro-inflammatory NO (eliciting nitrative stress protein modification) and cytokines (e.g., TNFα, IL1β) through the NFκB pathway, and NFκB activated iNOS. These pro-inflammatory molecules further activate the NFκB pathway and iNOS by a positive feedback loop [105]. Chronic neuroinflammation promotes pathologic protein modifications, e.g., the formation of toxic Aβ peptides and AβO, contributes to ERAD dysregulation, thus sustaining ER stress and the UPR. In addition to pro-inflammatory cytokines, protein modifications by S-nitrosylation, also sustain ER stress by (a) attenuating the UPR signalling via PERK and IRE-1; (b) increasing the proportion of activated Cdk5 kinase and consequently tau hyperphosphorylation with an increased burden for ER proteostasis; and (c) increased activity of Drp1 GTPase that promotes MITO fragmentation, reduced ATP production and neuronal death [105,140].

ER stress that leads to dysregulation of ERAD (e.g., due to Aβ peptides, mutant PS2 or tau overexpression), elicits Ca^2+^ release from ER to the MITO. Excessive Ca^2+^ influx to the MITO initiates the MITO apoptotic pathway as measured by MITO membrane depolarisation, cytochrome c release due to Bax translocation to mitochondria, and caspase-9 activation [142,143,144,145,146]. ER stress also releases Ca^2+^ into the cytosol, and increased cytosolic free calcium activates GSK3β thus promoting tau hyperphosphorylation and chronic inflammation. In summary, toxic forms of Aβ peptides and AβO establish a positive feedback loop between ERAD and MITO dysregulation (reduced ATP production, increased ROS) that ends in cell death [141]. A contributing factor to ERAD dysregulation in AD is the depletion of intracellular antioxidant glutathione (GSH) levels (presumably associated with the Aβ products elicited Ca^2+^ release from ER) measured in AD cell and animal models, and in patients with AD. Optimal intracellular GSH levels are sustained by nuclear factor-erythroid factor 2-related factor 2 (NRF2). PERK activation stimulates NRF2 expression, however, this effect is attenuated by an increased GSK3β activity in AD [141].

Numerus studies in animal models of AD provide ample evidence for a link between ERAD dysregulation and AD pathology, reviewed in [105,141,147,148]. For example, in the 5XFAD mouse model of AD, increased neuronal levels of BACE1 and Aβ accumulation were associated with eukaryotic translation initiation factor 2 subunit 1 (eIF2α) phosphorylation, due to loss of PERK inhibition [149]. Studies of ER stress and UPR in patients with AD provide inconsistent results due to post-mortem deterioration of mRNA and protein in post-mortem human brain samples [141,147].

**Figure 1:**
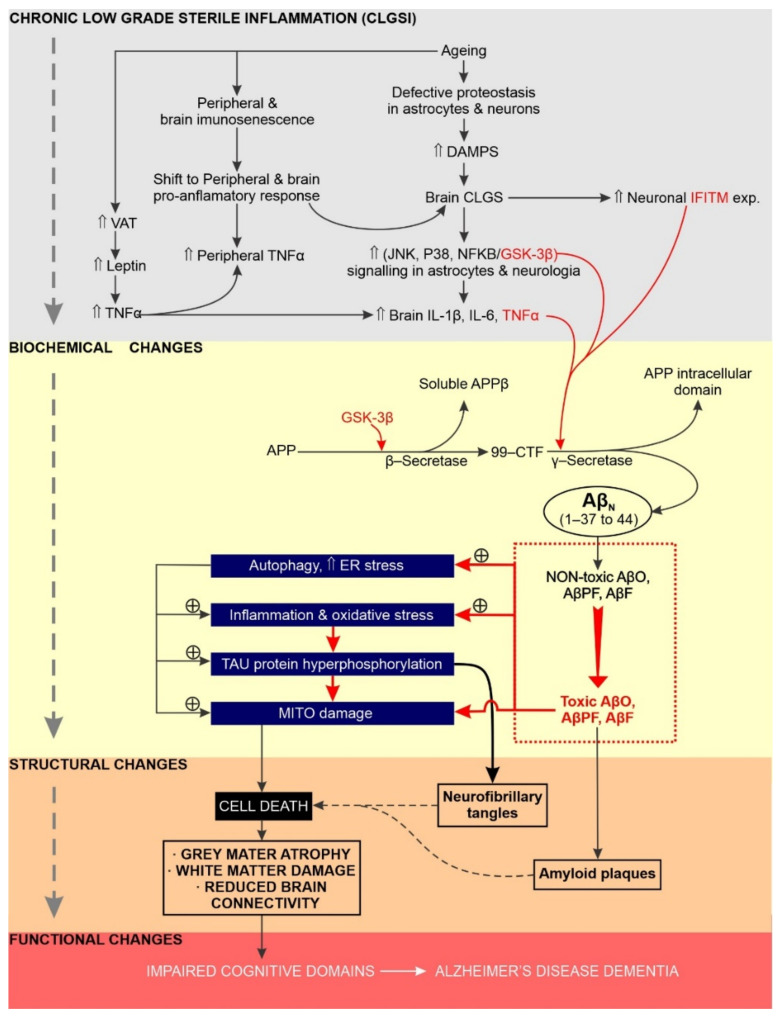
Chronic low grade sterile inflammation promotes AD-related changes in APP processing. Abbreviations: 99-CTF (99-amino acid membrane bound C-terminal fragment), AβF (amyloid β fibrils), Aβn (amyloid β peptides with 37 to 44 amino acid residues), AβO (amyloid β oligomers), AβPF (amyloid β protofibrils), APP (amyloid precursor protein), DAMPS (damage-associated molecular patterns), ER (endoplasmic reticulum), IFITM (interferon-induced transmembrane protein), IL -1β, -6, (interleukins -1β, -6), JNK (c-Jun N-terminal Kinase), MITO, NFκB/GSK3β (nuclear factor kappa-light-chain-enhancer of activated B cells/glycogen synthase kinase 3β), P38 (Mitogen-activated protein kinase 38), TNFα (tumour necrosis factor α), VAT.

The effects of SCI and chronic neuroinflammation on the initiation, sustainability and progression of AD associated dementia are summarised in Figure 1.

## 6. Physical Activity Delays Ageing-Related Changes

### 6.1. Human Studies

Physical activity at any level contributes to healthy ageing, delays cognitive and physical decline [150]. Ageing related memory deficits are correlated with a reduced functional connectivity within the anterior and posterior default mode network in the hippocampus. In healthy, randomly recruited individuals, a higher PA score is positively correlated: (a) with a reduction in negative age-related decreases in functional connectivity of posterior default-mode network, and (b) with increases in posterior cingulate cortex (PCC) grey matter volume, PCC perfusion, and (c) improved visuospatial task performance. These positive, brain ageing reducing effects on PCC were achieved with over a decade of PA. PCC functional connectivity is also reduced in the early stages of AD [151]. Meta-analysis of randomised, controlled trials, in healthy adults aged 50 and older, reported improved memory, executive function, auditory attention, cognitive speed, visual attention motor function and cardiorespiratory fitness after aerobic PA programmes [152,153]. Guidelines for healthy ageing recommend a personalised, PA regime that enables at least 150 min of moderate-intensity aerobic activity, or 75 min of vigorous-intensity aerobic activity combined with at least two days of muscle-strengthening activities per week [154]. Physical activity also (a) reduces TNFα activity (a pro-inflammatory cytokine that attenuates apoptosis), and (b) increases brain-derived neurotrophic factor (BDNF) activity in hippocampal and cortical neurons thus contributing to improved neuronal survival, learning and memory [13,155].

### 6.2. Effects of Physical Activity and Ageing on Proteostasis

Aging is associated with a reduced proteostasis efficiency, including among others a decreased autophagy and a reduced efficiency of the ubiquitin-proteasome system (as evidenced by an intracellular accumulation of dysfunctional proteins and organelles, misfolded proteins, increased conversion of misfolded proteins into toxic peptides and protein aggregates) that contribute to neurotoxicity, neurodegeneration, accelerated ageing process and a reduced life span [156,157,158,159]. Physical activity stimulates autophagy via AMP-activated protein kinase (AMPK) activation. Increased AMPK activity inhibits the target of rapamycin complex 1, a negative regulator of autophagy and a positive regulator of cellular protein production. Thus, the combined actions of an enhanced autophagy and a reduced cell protein burden delay the development and progression of neurodegeneration [160,161,162]. Activities of the autophagy and ubiquitin-proteasome system protein degradation pathways are coordinated. It has been suggested that the early post-exercise protein degradation is mediated mainly by the UPR and the late post-exercise protein degradation by autophagy [159]. For example, in human skeletal muscle, aerobic PA stimulates autophagy in a duration and intensity dependent manner [163,164,165]. Ageing also reduces the efficiency of the UPR; both endoplasmic reticulum (ER) protein folding and UPR protein degradation are reduced [166]. In human and animal studies, regular aerobic exercise seems to attenuate ER stress in middle-aged and old subjects [166,167,168]; however, the relationship among UPR activation, exercise and aging has to be further investigated in more detail, especially in human subjects [166].

## 7. Physical Activity Attenuates Expression of Pro-Inflammatory Markers

### 7.1. Overview

Obesity, by inducing local hypoxia in the enlarged, VAT, stimulates sustained release of pro-inflammatory mediators (e.g., TNFα) from resident adipose tissue macrophages. Long-term exercise, i.e., more than 16 weeks, attenuates expression of pro-inflammatory markers by reduction in the size of adipose tissue cells [169,170]. Compared to hypocaloric diets, exercise is more effective in reducing adipose tissue mass while conserving the bodies weight [171].

During exercise, the expression of skeletal muscle mRNA PGC1α is increased via AMPK activation, and returns to baseline values after exercise [172,173]. Increased mRNA PGC1α expression is assumed to attenuate expression of pro-inflammatory cytokine TNFα and oxidative stress-mediating genes in vascular endothelial cells, and to change the balance in favour of anti-inflammatory (M2) skeletal muscle macrophages [169]. Increased levels of TNFα stimulate muscle catabolism via NFkB signalling pathway that promotes ubiquitin conjugation of muscle proteins and their proteasome degradation [169]. Depression symptoms are associated with increased levels of TNFα; in students, moderate intensity, continuous PE decreased depressive symptoms, perceived stress and TNFα levels compared to healthy students with no exercise [174]. Moderate intensity, continuous PE had a similar effect on pro-inflammatory IL1β levels that was not significant [174].

Increased PGC-1α levels in contracting muscle fibbers also stimulate the release of irisin, produced from fibronectin type III domain-containing protein 5 (FNDC5) in myocytes. Irisin crosses the BBB, attenuates brain neuroinflammation and improves hippocampal memory and learning function by increasing expression of BDNF in microglia and astrocytes. BDNF has anti-inflammatory effects by attenuating NFκB, GSK3β, p38 and JNK activity in microglia and astrocytes, thus reducing the release of pro-inflammatory cytokines of IL6 and IL1β in the brain [175]. Irisin also decreases the expression of pro-inflammatory cyclooxygenase-2 and AKT phosphorylation [175].

FNDC5 is also expressed in the hippocampus [176,177], and this brain expression could have an AD preventive effect. In vitro, FNDC5 binds to a specific domain between β- and α-secretase APP cleavage sites, thus reducing Aβ40 and Aβ42 formation [178]. In an animal model, the down regulation of brain FNDC5/irisin attenuated long-term potentiation and memory formation, while restored FNDC5/irisin brain levels improved synaptic plasticity and memory [179].

Animal studies explain the association between inflammation, depression and PE. Skeletal muscles metabolise kynurenine (a product of tryptophan metabolism) into kynurenic acid thus reducing the amount of kynurenine that crosses the BBB and induces depression. The conversion to kynurenic acid is enhanced by increased PGC-1α expression [180,181].

Physical exercise leads to a transient, moderate level, release of the anti-inflammatory skeletal muscle cytokine IL6; this cytokine inhibits the release of TNFα and stimulates the release of anti-inflammatory IL1 receptor antagonist in leukocytes and lymphocytes [182]. Thus, transient and moderate increases of IL6, released from myocytes, elicit in monocytes or macrophages an anti-inflammatory response, by a nuclear factor kappa-light-chain-enhancer of activated B cells (NFκB) independent signalling pathway, (i.e., with an increased production of IL10 and IL1RA). High levels and/or long term release of IL6 from macrophages elicits a pro-inflammatory response in monocytes or macrophages via the NFκB signalling pathway [183]. In summary, IL6 contribution to inflammation and muscle proteostasis is time and concentration dependent; transient and moderate increases of IL6 stimulate myogenesis and an anti-inflammatory response, chronic and large increases of IL6 promote a pro-inflammatory response in immune cells (via NFκB signalling pathway) and muscle wasting via STAT3 signalling [184].

### 7.2. Animal Studies

Animal studies on AD model rodents demonstrate the beneficial role of PE in attenuating the pro-inflammatory markers, the detrimental effects of neuroinflammation and the progression of AD. Treadmill exercise (30–60 min/day, 5–7 consecutive days, 1–12 weeks) attenuated cognitive deficits, decreased TNFα levels, astrocytosis and brain Aβ deposition [89,185]. Therefore, the beneficial effect of PE in animal AD models was evidenced after 1 week and was not transient, could be sustained for 12 weeks.

### 7.3. Human Studies

A meta-analysis of 18 observational or interventional studies, in healthy adults age 18–65, concluded that moderate exercise, or high intensity exercise with resting periods, offered an optimal balance between the benefits of physical training and the minimal risk of muscle injury with chronic inflammation [186]. The effects of 24 weeks combined aerobic and resistance training on TNFα levels was evaluated in 48 healthy young men (average age 31). TNFα levels were significantly reduced only with alternate day aerobic and resistance training, compared to the control inactive group or same day aerobic and resistance training group. The decrease in abdominal fat mass correlated with reduced blood levels of monocyte chemoattractant protein 1, leptin and resistin [187]. In male and female adults over 60 years, low PA is associated with a reduced life span from inflammatory diseases other than cardiovascular disorders or cancer [188].

Several meta-analyses of randomised, controlled trials (obese or non-obese male and female participants, participants’ age between 50–89 years) concluded that long-term PA (at least 3 times/week, for more than 12 weeks) attenuated pro-inflammatory markers (e.g., TNFα, C-reactive protein) probably due to a reduction in excess adipose tissue [189,190,191,192,193,194].

## 8. Physical Activity Modulates Adaptive Immunity

Regular, structured PE, provided it develops and sustains cardiorespiratory fitness, improves efficiency of adaptive immune system in human across all ages. Animal and human studies support the hypothesis that PE improves adaptive immunity by preventing the excessive accumulation of memory T lymphocytes in the body. Naïve T cells (e.g., CD4+ helper cells, CD8+ cytotoxic cells) circulate between the blood and the lymphatic system until they come into contact with antigens (on antigen presenting cells) recognised by naïve T receptors; this contact transforms naïve T cells into activated T cells that further differentiate to memory T lymphocytes. Memory T lymphocytes have a lower antigen activation threshold, a higher rate of proliferation and a better peripheral tissue and secondary lymphatic tissue penetration than naïve T cells, thus responding more quickly, in more tissues and more forcefully to a repeated antigen challenge. The process of PE promotes redistribution of CD4+ and CD8+ antigen-experienced memory T lymphocytes from the lymphatic tissue to the blood vessels, followed by migration of memory T lymphocytes to the peripheral tissue where they are eliminated by contact with pro-apoptotic molecules (ROS, cytokines and glucocorticoids). This reduction in the number of memory T lymphocytes is assumed to trigger a compensatory increase in the number of naïve T lymphocytes (T cells not yet in contact with a specific antigen) by a negative feedback loop governing the ratio of memory to naïve T lymphocytes [195].

## 9. Physical Activity Attenuates AD Neuroinflammation

### 9.1. Animal Studies

Animal studies on AD model rodents demonstrate the beneficial role of PE in attenuating AD pathology related changes. The pooled effects of treadmill exercise (30 min/day, 5 consecutive days, 1 week to 5 months) or swimming training (20–60 min/day, 5 days/week, 8 weeks) were: (a) attenuated Aβ-induced cognitive deficits; (b) improved adult hippocampal neurogenesis; (c) reduced hippocampal neuroinflammation (measured by reduced levels of TNFα, IL1β, and IL6); (d) attenuated levels of indoleamine-2,3-dioxygenase stimulated neurotoxic tryptophan catabolites; (e) partial reversal of Aβ-attenuated levels of BDNF, glial cell line-derived neurotrophic factor, nerve growth factor and neurotrophin-3 brain levels; and (f) reduced hippocampal tau phosphorylation and Aβ deposition in trained animals, compared to control untrained animals [89,196,197].

In various AD animal models, PE reduces hippocampal inflammation and the further hippocampal Aβ products deposition by: (a) an up-regulation of disintegrin and metalloproteinase 17 mRNA and down-regulation of BACE1 mRNA in ageing rats [198]; (b) an attenuation of brain GSK3α/β and/or CDK5 activity [197,199,200,201,202,203,204,205,206,207]; (c) a reduced APP phosphorylation [197]; (d) an attenuated activity of tau kinases that reduces tau phosphorylation and also reduces tau kinase mediated APP phosphorylation and γ-secretase activation [197,208]; (e) an attenuation of neuroinflammation stimulated indoleamine-2,3-dioxygenase activity by the anti-inflammatory cytokine IL10 [196,209], and (f) an attenuated phosphorylation of pro-inflammatory p38 and JNK molecules, due to a reduced MAPK and NFκB signalling [89].

### 9.2. Human Studies

There is a lack of controlled, randomised studies that evaluate the effect of PE on systemic and brain pro-inflammatory markers in patients with AD. A two-months aerobic exercise regime improved quality of life and psychological wellbeing parameters, and reduced systemic pro-inflammatory markers (e.g., TNFα) in patients with AD (age 67 to 75 years, male and female participants) [210]. The effect of exercise on inflammation markers in AD patients was recently evaluated in 16 weeks long, randomized controlled trial with 198 participants (average age 70 years, male and female participants), distributed among control, moderate and high exercise groups. The outcomes of PE were: (a) a small increase in plasma IL6 after PE, (b) a reduced IFNγ concentrations in APO ε4 carriers, (c) no significant effect on cerebrospinal fluid (CSF) levels of cytokines IL-10, -13, -2, -6, -8, and TNFα, and (d) the marker for myeloid cells 2 trigger receptor (measuring microglial activation) was significantly increased in CSF. The recommendations, for future evaluations of exercise-elicited effects on pro-inflammatory markers in patients with AD, were: to evaluate the duration and type of PE, to increase the number of participating patients, and to stratify the effects of exercise protocol on different stages of AD, from pre-clinical to severe AD [211].

## 10. Physical Activity Attenuates AD Progression

### 10.1. Muscle Activity Modulates Cognition via Muscle-Brain Interactions

The intensity of PE leads to a proportional increase in the release of adiponectin from adipose tissue, and IGF1 from the liver and contracting muscles. These signalling molecules cross the BBB and modulate brain activity. Adiponectin brain actions support neurogenesis, learning, memory formation and ameliorate depression-like behaviour [176]. IGF1 supports normal cognition directly by upregulating hippocampal BDNF expression and adult neurogenesis, and indirectly by increasing Aβ peptide brain clearance, stimulating Aβ peptide degradation by insulin-degrading enzyme (IDE) and increasing cellular uptake and lysosomal degradation of Aβ peptide [176,212]. Physical exercise in mammals also stimulates the release of skeletal muscle myokines cathepsin B and irisin (also discussed in Section 7.1). Both of them enhance neurogenesis, learning, memory and depression-free mood by stimulating BDNF brain expression [176].

Irisin inhibits the binding between Aβ oligomers and neurons, thus preventing eIF2α phosphorylation (the phosphorylated form acts as an inhibitor of its own guanine nucleotide exchange factor) and inhibition of protein synthesis [213]. In non-demented humans, the levels of CSF irisin increase with ageing. Patients with AD have normal irisin plasma levels, concomitant with reduced CSF irisin levels. Hippocampal FNDC5/irisin is reduced in moderate-to-late AD, but not in MCI [213]. A recent study reported that CSF irisin levels were positively correlated with CSF BDNF and Aβ42 CSF levels, and with MMSE scores, but not with CSF total tau. Therefore, decreased CSF irisin and BDNF levels do not seem to be associated with total tau but with brain amyloid pathology. Compared to non-demented controls, patients with AD had reduced CSF levels of BDNF and Aβ42, increased levels of CSF total tau, and lower cognitive scores [214].

Increased BDNF brain expression is also elicited by an increased sympathetic nervous system activity and elevated blood concentration levels of ketone bodies during PE [176]. BDNF attenuates Aβ peptide toxicity on neurons, promotes synaptic plasticity by increasing the strength of synaptic connections, promotes LTP and by extension memory formation, learning and cognition, therefore is essential for normal hippocampal neurogenesis and development of hippocampal neural circuits [215]. Interventions to increase brain BDNF in human could improve learning and memory, ameliorate AD pathology and mood disorders [216,217,218,219,220,221].

### 10.2. Human Studies on Old Age Health Subjects

Changes in hippocampal volume, in response to different levels of PA, can occur within weeks. In young to middle-aged adults, a six-week aerobic training exercise intervention transiently increased the hippocampal volume (due to an increase in hippocampal myelination). This observed increase was reversed after six weeks without aerobic exercise [222].

Memory function and plasma values of factors BDNF, IGF1, VEGF or platelet-derived growth factor C were measured before and after a 3-month aerobic exercise regime in 40 humans, age 60–77 years. Although the aerobic exercise regime improved memory function, there were no concomitant changes in the measured plasma values. Explanations given for the observed discrepancy were: a high intra-individual variability of base plasma values, a low number of participants, diurnal variation of measured factors due to sex and/or other interfering metabolic processes (e.g., food intake) [223].

56 healthy elderly participants, male and female, (average age 68) were involved in 12 weeks randomised physical training study (high resistance training only (80% of one repetition maximum (1RM), low resistance training only (20% 1RM), or mixed low resistance training (20% and 40% 1RM)). BDNF levels were increased in males only of the mixed low resistance training group [224].

The lack of PA effects in women [224] is consistent with the study results where lifelong aerobic exercise did not protect elderly women against age-related increases in circulating pro-inflammatory markers or muscle inflammation and that in elderly women the preparedness to handle loading stress was not preserved by lifelong exercise [225].

A one-year, randomised and controlled, moderate-intensity aerobic exercise training regime, with 120 participating male and female adults (age 55–80), increased the volume of the anterior hippocampus by 2%, improved spatial memory and increased BDNF serum levels compared to control, group. The observed increase in volume was sufficient to compensate for the expected, age-related 1–2% percent volume decrease in older adults without dementia, thus also reducing the risk for cognitive impairment [226].

### 10.3. Human Studies on Persons with AD

Cognition and molecular biomarkers were evaluated in two subgroups of nondemented persons with a family history of Alzheimer’s disease; subgroup + APO ε4 (with APO ε4 genotype) and subgroup—APO ε4 (without the APO ε4 genotype allele), and compared to their senior functional physical fitness test values. The + APO ε4 subgroup had a lower cognitive score, when performing cognitive tasks with a higher visuospatial working memory load, compared to the—APO ε4 subgroup. There were no significant changes in the levels of molecular markers IL1β, BDNF, Aβ40 and Aβ42 between the two subgroups. Compared to the + APO ε4 subgroup, the—APO ε4 subgroup had a better cardiorespiratory fitness score, and this difference was positively correlated with the higher cognitive fitness in the—APO ε4 subgroup [177].

The pooled positive effects of PA on attenuating cognitive decline in AD patients, reported in several randomised controlled trails, are: a reduced decline in daily living activities, an improved score on neuropsychiatric symptoms, immediate and delayed memory improvements and exercise-related gains in cardiorespiratory fitness that are associated with improved memory performance and reduced hippocampal atrophy [12,227,228,229,230].

The effects of PA or PE in human on attenuating MCI, on preventing the risk of AD or attenuating cognitive impairment in patients diagnosed with AD are summarised in recent (between 2017–2022), randomised controlled trials and prospective studies presented in Table 1. The 2016 trial, on the effect of of PA on inflammation in patients with AD, is also included in the table, since between 2016 and 2022, only two randomised controlled trails were published on the effect of PA on inflammation in patients with AD.

Improvements in protocols of future studies, to develop effective treatment interventions for delaying the progression of AD with PA or PE, should include:

(a) data stratification by sex on the effects of physical activity-related improvements in cognition;
(b) measures to improve participants’ compliance with supervised training;
(c) an increased number of participants;
(d) extending the trail’s duration to 12 months or more;
(e) robust inclusion and exclusion criteria for study participant selection; and
(f) use of a standardised PA or PE regime and a comprehensive evaluation protocol for measuring physical activity-related improvements in cognition.

Moderate endurance and resistance training PE improved quality of life, cardiovascular fitness and motor function without extending life expectancy in patients with ALS [238,239,240]. Aerobic and resistance PE, combined with training to improve balance, gait and coordination, improved quality of life motor function (balance, gait, reduced risk of falls), sleep and cognition in patients with PD [241,242,243]. Exercise may cause motor neuron injury in ALS patients with a risk-genotype [244]. Further clinical trials are necessary to develop personalised, disease progression tailored PE treatment interventions for these patients.

### 10.4. Animal Studies

The beneficial effects of regular PE are established for many acute and chronic brain disorders [245]. The molecular biology bases for attenuation of AD progression in rodent models by physical activity are: (a) PA stimulates release of brain BDNF and (b) the β-secretase (BACE1) promoter that stimulates expression of β-secretase, the rate limiting enzyme of the amyloidogenic pathway producing Aβ peptides in the neurons, has a NFκB biding site. Activation of the NFkB signalling pathway promotes increased levels of pro-inflammatory cytokines and free radicals [245]. BDNF modulates the balance between the non-amyloidogenic processing of APP and the amyloidogenic processing of APP in neurons. Increased BDNF levels shift the balance of APP processing to the non-amyloidogenic APP pathway, by upregulating α-secretase activity and subsequently increasing production of the sAPPα fragment [246]. Decreased BDNF levels promote sequential APP processing by β- and γ-secretase with the end result of increased, toxic levels of Aβ peptide with 42 amino acid residues (Aβ42) and its toxic oligomers [247,248,249]. In contrast to normal, physiologic Aβ peptides levels, increased, toxic levels of Aβ peptides stimulate β-secretase expression by the NFκB transcription factor protein [250].

The pooled results of regular PA, recently studied in a variety of AD rodent models (mice overexpressing a mutant form of APP or a human presenilin 1 and a chimeric amyloid precursor protein; streptozotocin or Aβ peptide brain infusion elicited AD), are: (a) the preferred training method was forced treadmill running, exercise intensity at about 50% of VO2 max; and (b) the training duration was 20–60 min/day, 5–7 days per week, between 1 week and 5 months. Compared to AD model rodents without treadmill exercise, the measured changes in the exercised AD animals were: (a) the histologically and electrophysiologically observed enhanced synaptic plasticity, the reduced spatial learning and memory impairment; (b) the reduced levels of APP, BACE-1 and soluble Aβ40–42 proteins in the cortex/hippocampus; (c) the increased expression of Aβ clearance proteins (neprilysin, IDE, matrix metalloproteinase-9, low density lipoprotein receptor-related protein 1 and 70 kilodalton heat shock protein); (d) the reduced expression of RAGE mRNA; (e) the increased hippocampal volume with an increased number of neurons, the improved MITO function; (f) the reduced hippocampal neuroinflammation, as evidenced by the decreased levels of TNFα and IL1β and the reduced number of astrocytes; (g) the attenuated ER stress, measured by the down-regulation of ATF6 and spliced X-box binding protein 1, and by the reduced activation of JNK and p38 in the hippocampus; and (h) the above mentioned positive, regular PA effects were also present at the amyloid plaque stage in the brain and correlated with exercise intensity [24,89,185,251,252,253,254,255,256].

The effects of PA or PE on cognition and brain pathology in animal models of AD and cerebral amiloid pathology animal model (CAA) are summarised in recent (between 2017–2022) studies presented in Table 2.

## 11. Conclusions

**Figure 2:**
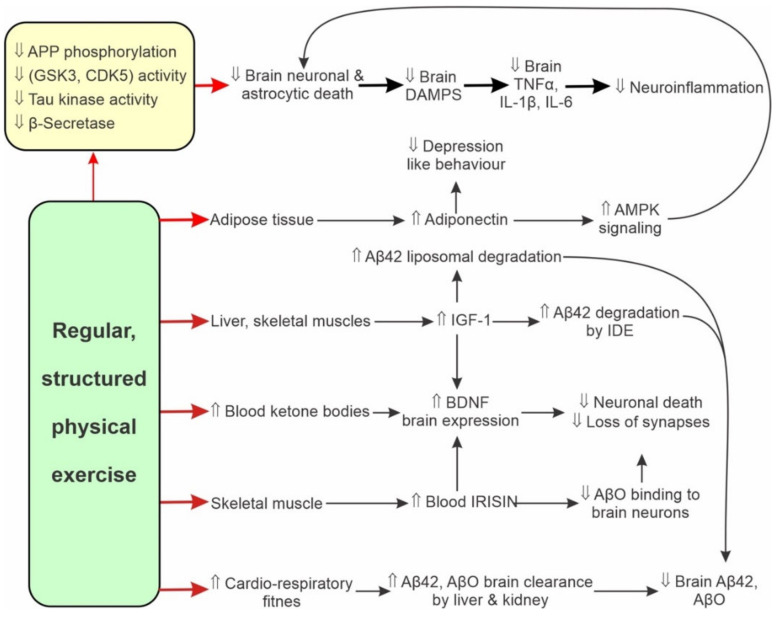
Modulation of neuroinflammation and Aβ peptides processing by regular, structured physical exercise. Abbreviations: Aβ42 (amyloid β peptide with 42 amino acid residues), AβO (toxic amyloid β oligomers), AMPK (5′ AMP-activated protein kinase), DAMPS (damage-associated molecular patterns), BDNF (brain-derived neurotrophic factor), CDK5 (cyclin dependent kinase 5), DAMPS (damage associated molecular patterns), GSK3 glycogen synthase kinase 3), IDE (insulin-degrading enzyme), IGF1 (insulin-like growth factor 1), IL -1β, -6, (interleukins -1β, -6), TNFα (tumour necrosis factor α).

Regular, structured PE should be considered as a supplemental intervention for attenuating the progression of AD in human by improving cardiovascular fitness and reducing systemic and brain inflammation. Animal studies consistently report that PE improves cardiovascular fitness and attenuates AD-promoting processing of APP and neuroinflammation, facilitates brain clearance of toxic Aβ peptides and oligomers and promotes brain connectivity and nerve cell viability (Figure 2). Further studies in human are necessary to develop optimal, personalised protocols to take full advantage of the beneficial effects of PE that promote cardiovascular fitness, attenuate systemic inflammation, stimulate brain Aβ peptides brain catabolism, delay immunescence and support brain clearance of Aβ peptides and their catabolism in peripheral organs.

## Figures and Tables

**Table 1:** Effect of physical activity (PA) or physical exercise (PE) on attenuating MCI, preventing the risk of AD or attenuating cognitive impairment in patients diagnosed with AD. PA refers to any movement that is carried out by the muscles that requires energy. PE is a planned, structured, and repetitive PA with the objective to improve or maintenance of physical fitness. Only two randomised controlled trails, reporting the effect of PA on inflammation in patients with AD, were published between 2016–2022.

Study Design	Participants	Result	Refs.
Systematic review and meta-analysis of observational prospective studies and randomised controlled trials	Participants from 243 observational prospective studies and 153 randomised controlled trials	21 most important evidence-based suggestions for life-course practices to prevent AD were identified and divided by the level of evidence (into levels A and B) and strength of suggestion (into class I and class III). PA was classified among the 21 most important evidence-based suggestions for life-course practices to prevent AD into level B, class I.	[231]
Randomised controlled trial	Men and women (n = 106) randomised in control (n = 51) and treated groups (n = 55), aged 60 years or older, with MCI or subjective memory complaints (SMC) and at least one CVR factor (physical inactivity, obesity, hypertension, heart disease, type II diabetes, smoking, hypercholesterolemia)	After 24-months, the home based PA programme improved CVF and leg strength; cognition was not evaluated.	[232]
Meta-analysis of randomised control trials	673 subjects with AD in 13 randomized controlled trials, treated to different quantities of physical activity and exercise interventions, were included.	PA and PE improved cognition of older adults with AD. High frequency PA and PE interventions did not have a greater effect on cognition compared to low frequency interventions.	[233]
Large prospective observational study	Two prospective studies. First study on 197,685 long-distance skiers to compare the incidence of vascular dementia (VAD) or AD to matched individuals from the general population (n = 197,684 participants) during 21 years of follow-up. Second study evaluated the association between self-reported PA and the incidence of VAD and AD in 20,639 participants.	PA in midlife reduced the incidence of VAD. There was no significant association between PA and the risk of subsequent development of AD.	[234]
Randomised controlled trail	198 male and female patients, average age 71 and 70 for control and exercise groups, with clinically diagnosed AD by the NINCDS-ADRDA criteria and an MMSE >19.	16 weeks of moderate-to-high PE attenuated plasma INFγ in APO ε4 patients with AD.	[211]
Randomised trial	1260 people, age (60–77 years), at risk for dementia were randomized 1:1 to multidomain intervention (diet, PE, cognition (evaluated by the Mini–Mental State Examination, and vascular risk management) and regular health advice).	The 2-year study intervention improved overall cognitive performance (measured with an extended Neuropsychological Test Battery (NTB) and was beneficial regardless of participants’ sex, age, income, cognition, body mass index, blood pressure, cholesterol, fasting glucose, overall cardiovascular risk, and cardiovascular comorbidity	[235]
Randomised controlled trail	494 male and female participants with dementia (measured by the Alzheimer’s disease assessment scale-cognitive subscale), average age 77 years.	12 months of moderate to high intensity aerobic and strength PE training programme did not attenuate cognitive impairment nor improve activities of daily living in people with mild to moderate dementia. Physical fitness was improved.	[236]
Meta-analysis of randomised controlled trilas	Older adults with MCI or dementia from 10 randomized controlled trials that evaluated the effect of a combined cognitive-physical intervention on cognition.	Combined cognitive-physical interventions were equally beneficial to older adults with MCI or with dementia: there was a small-to-medium positive effect on global cognitive function and a moderate-to-large positive effect for activities of daily living.	[237]
Pilot, randomised controlled trail	76 male and female participants over 55 years of age, mean age 73, with MCI or dementia with etiologic diagnosis of probable AD based on clinical and cognitive test results.	26 weeks of supervised PE improved cardiorespiratory fitness associated with a modest improvement in functional ability (measured by the Disability Assessment for Dementia). The was no measurable improvement in memory, executive function, or depressive symptoms. Improved cardiorespiratory fitness was positively correlated with change in memory performance and reduced bilateral hippocampal atrophy.	[230]
Randomised controlled trail	40 male and female patients with AD, age 65–75, were divided into control (no training intervention) and treadmill aerobic exercise group. Both groups were evaluated for TNF-α, interleukin-6 IL-6, Rosenberg Self-Esteem Scale, Beck Depression Inventory, Profile of Mood States and SF-36 health quality of life before and at the end of the study.	2-moths of PE improved quality of life, attenuated systemic inflammation markers and psychological wellbeing in patients with AD.	[210]

**Table 2:** Effect of physical activity (PA) or physical exercise (PE) on cognition in animal models of AD and CAA. Abbreviations: CAAam (cerebral amiloid pathology animal model); MT (constant speed motorised treadmill); (FADam) dominantly inherited familial AD animal model; (LOADam) late onset AD animal model; (R) adult rats; (M) adult mice (M); WT (wild type).

Animal Model, Age at Start of Experiment	PA or PE Design	Result, Compared to Sedentary Animal Models of AD and CAA	Refs.
CAAam (Tg-SwDI male and female M); C57BL/6 WT male and female M, age 4 months	Voluntary wheel running PA, wheel availability 1–12 h/day, 5 days/week, 8 consecutive months	PA improved motor function, reduced anxiety-like behaviour and attenuated neuroinflammation markers TNFα and IL6 but not vascular amyloid β accumulation.	[257]
FADam, 5xFAD male M; WT JAXC57BL/6J male M, age 6 weeks	Voluntary wheel running PA, wheel availability 24 h/day, 7 days/week, 6 consecutive months	PA mitigated Aβ pathology related cognitive deficits in spatial learning, memory and exploration activity with a temporal association to increased hippocampal glial fibrillary acid protein (GFAP) immunoreactivity and the number of GFAP-positive astrocytes, increased astrocytic brain-derived neurotrophic factor and restoration of postsynaptic protein PSD-95. Voluntary PE did not attenuate brain neuroinflammation markers.	[258]
FADam, 5xFAD female M; age 9–12 weeks	Voluntary wheel running PA, wheel availability 24 h/day, 7 days/week, 4 consecutive weeks	PA did not attenuate neuroinflammation markers (total amount of neuroglia in hippocampus, cytokine levels, levels of NLRP3), nor improve motor learning or reduce insoluble Aβ brain content.	[259]
FADam, APP/PS1 male and female M, age 12 months	MT PE, 20 min/day, 5 days/week, 4 consecutive months	PE mitigated Aβ pathology related cognitive deficits in spatial cognition with a temporal association to increases in spinophilin-immunoreactive puncta numbers in hippocampal areas. The effect of PE on neuroinflammation markers was not evaluated.	[24]
FADam, 5xFAD female M, age 9–12 weeks	Voluntary wheel running PA, wheel availability 24 h/day, 7 days/week, for 24 consecutive weeks.	PA did not mitigate Aβ pathology related cognitive deficits in object or working memory, nor synaptic proteins PSD-95 and synaptophysin contents, Aβ brain content or hippocampal Aβ42 concentration. The effect of PE on neuroinflammation markers was not evaluated.	[234]
FADam, APP/PS1 male M; WT C57BL/6 male M, age 3 months	MT PE, 45 min per day, 5 days/week, 3 consecutive months	PE mitigated Aβ pathology related deficits in cognition associated hippocampus, with reduced Aβ plaques and soluble Aβ forms, decreased β-site amyloid precursor protein-cleaving enzyme 1 and presenilin-1 expression, downregulated expression of GRP78, and inhibited activation of PERK, eIF2α, and ATF4. The effects of PE on neuroinflammation markers and animals’ cognitive behaviour were not evaluated.	[260]
FADam, APP/PS1 male M; WT C57BL/6 male M, age 6 months	MT PE, 20 min per day, 5 days/week, 4 consecutive months	PE mitigated Aβ pathology related cognitive deficits in special learning and memory abilities with a temporal association to increased hippocampal volumes and increased number of hippocampal neurons. The effect of PE on neuroinflammation markers was not evaluated.	[256]
FADam, APP/PS1 M; WT C57BL/6 M, age 5 months	MT PE, 30 min per day, 6 days/week, 5 consecutive months	PE mitigated Aβ pathology in cognition asociated hippocampus and neocortex (attenuated Aβ area fraction, plaque number and size and decreased levels of insulin-degrading enzyme and receptor for advanced glycation end products). Also, PE increased neuronal density, attenuated activation of astrocytes and decreased β-site amyloid precursor protein cleaving enzyme 1 and presenilin 1 levels. The activity of non-amyloidogenic APP pathway was increased. The effect of PE on animals’ cognitive behaviour was not evaluated. Controlled PE had a possible inhibitory effect on neuroinflammation by supressing any numerical and morphological conversions of microglia and by reducing the total number of astrocytes and the number of astrocytes associated with Aβ pathology.	[261]
LOADam, icvi. of streptozotocin, Wistar male R, age 6 weeks	MT PE, 1 h/day, 5 days/week, 24 weeks (8 weeks before icvi and consecutive 12 weeks after)	PE mitigated Aβ pathology related cognitive deficits in spatial cognition and willingness to explore with a temporal association to positive changes in MITO oxygen consumption endpoints of synaptosomal and non-synaptosomal brain mitochondria. The effect of PE on neuroinflammation markers was not evaluated.	[255]
LOADam, icvi. of Aβ42 peptide, Wistar male R, age 7 weeks	MT PE, two, 15 min sessions/day in weeks 1 and 2, increased to 3 sessions/day in week 3 and 4 sessions/day in week 4	PE prevented Aβ pathology associated increase in levels of APP, BACE-1 and Aβ proteins in hippocampal areas (associated with cognitive functions). The effects of PE on neuroinflammation markers and animals’ cognitive behaviour were not evaluated.	[245]
LOADam, ihi. of Aβ42 peptide, C57BL/6N male M, age 8 weeks	MT PE, 30/day, 7 consecutive days	PE mitigated Aβ pathology related cognitive deficits in object recognition and spatial cognition with a temporal association to hippocampal increased adult neurogenesis, decreased inflammatory cytokine levels and decreased astroglial cell density. Also, PE partly normalised MAPK signalling (i.e., attenuated JNK and P38 phosphorylation).	[89]
LOADam, icvi. of Aβ42 peptide, Swiss Albino male M, age 3 months	swimming PE with weights attached to the proximal portions of animal’s tail, duration progressively increased from 20 to 60 min/day, 5 days/week, 8 consecutive weeks	PE mitigated Aβ pathology related cognitive deficits (memory impairment and depressive/anxiety-like behaviour) with a temporal association to inhibition of inflammation/indoleamine-2,3-dioxygenase activation and up-regulation of neurotrophic factors in brain.	[196]

## Data Availability

Not applicable.

## Abbreviations

1RM	one repetition maximum
99-CTF	99-amino acid membrane bound C-terminal fragment
A-AMP	anionic antimicrobial peptide
Aβ40,42	amyloid β peptide with 40 or 42 amino acid residues
AβF	amyloid β fibrils
AβO	soluble amyloid β oligomers
AβPF	amyloid β protofibrils
AD	Alzheimer’s disease
AKT	protein kinase B
AMPK	5′ AMP-activated protein kinase
APH-AD	Antimicrobial Protection Hypothesis of AD
APO	apolipoprotein
APP	amyloid precursor protein
ATF6	activating transcription factor 6
ATP	adenosine triphosphate
BACE1	β-secretase
BBB	blood-brain barrier
BDNF	brain-derived neurotrophic factor
CAA	cerebral amiloid pathology
CNS	central nervous system
CSF	cerebrospinal fluid
DAMPS	damage associated molecular patterns
DM	diabetes mellitus
eIF2α	eukaryotic translation initiation factor 2 subunit 1
eIF2B	guanine nucleotide exchange factor (GEF) for its GTP-binding protein partner eIF2
EOAD	early onset Alzheimer’s disease
ER	endoplasmic reticulum
ERAD	endoplasmic-reticulum-associated protein degradation
FAD	familial Alzheimer’s disease
FNDC5	fibronectin type III domain-containing protein 5
GSH	glutathione
GSK3β	glycogen synthase kinase 3β
IDE	insulin-degrading enzyme
IFITM	interferon-induced transmembrane protein
IGF1	insulin-like growth factor
IL	interleukin
iNOS	inducible nitric oxide synthases
JAK	cytokine-activated Janus kinase
JNK	c-Jun N-terminal Kinase
LBD	Lewy body dementia
LOAD	late onset Alzheimer’s disease
LRP1	low density lipoprotein receptor-related protein 1
LTD	long-term depression
LTP	long term potentiation
MCI	mild cognitive impairment
MITO	mitochondrial
NAD^+^	nicotinamide adenine dinucleotide
NFκB	nuclear factor kappa-light-chain-enhancer of activated B cells
NFT NLRP3	neurofibrillary tangles NLR family pyrin domain containing 3 protein
NK	natural killer
NMDAR	N-methyl-D-aspartate receptor and ion channel
NRF2	Nuclear factor-erythroid factor 2-related factor 2
P38	mitogen-activated protein kinase 38
PA	physical activity
PAMPS	pathogen associated molecular patterns
PE	physical exercise
PCC	posterior cingulate cortex
PERK	protein kinase R-like endoplasmic reticulum kinase
PGC1α	peroxisome proliferator-activated receptor-γ coactivator
PI3K	phosphatidylinositol 3-kinase
PKA	protein kinase A
PKB	protein kinase B
PKC	protein kinase C
PRRS	pattern recognition receptors
PS1	presenilin-1
PS2	presenilin-2
RAGE	receptor for advanced glycation end products
ROS	reactive oxidative species
SASP	senescence-associated secretory phenotype
SAT	subcutaneous adipose tissue
SCI	systemic low-grade chronic inflammation
STAT3/5	signal transducers and activators of transcription 3 and 5
TLR	toll-like receptor
TNFα	tumour necrosis factor α
UPR	unfolded protein response
VAD	vascular dementia
VAT	visceral adipose tissue
VEGF	vascular endothelial growth factor
